# Comment on “Long-Term Dietary Changes after Diagnosis of Rheumatoid Arthritis in Swedish Women: Data from a Population-Based Cohort”

**DOI:** 10.1155/2019/1939686

**Published:** 2019-01-02

**Authors:** Velammal Petchiappan, Preethi Priyadarshini, V. N. Nagaprabu

**Affiliations:** ^1^Department of Medicine, PSG Institute of Medical Sciences and Research, Coimbatore, India; ^2^Sakthi Rheumatology Centre, Coimbatore, India

We read with great interest the article published in International Journal of Rheumatology, “Long-Term Dietary Changes after Diagnosis of Rheumatoid Arthritis in Swedish Women: Data from a Population-Based Cohort” by Cecilia Lourdudoss et al. [1]. The role of diet in rheumatoid arthritis (RA) remains an ever-burning question for both the patient and the treating physician and, while it is quite acceptable for the lay person to think that the occurrence of the arthritis is influenced by the diet, unfortunately the scientific community is yet to produce a definite answer.

In a systematic review of dietary intervention studies in RA, it was concluded that the effect of various dietary manipulations on the disease is uncertain [2]. The potential risks associated with such intervention were also highlighted in that review. The present study is among the important observations in this field and the authors give specific dietary recommendations for patients with RA in the future. The authors noted that there was no change in dietary pattern in RA patients, whereas those without RA made certain changes to their dietary pattern during the study period. This might be because of the relatively small RA population when compared to the controls in this cohort. The duration of the disease, the disease activity, and the occurrence of comorbid illness also were not taken into consideration.

We did a study that assessed the role of diet in RA patients and its impact on arthritis-related symptoms as perceived by the patients themselves, using a simple questionnaire in their own language [3]. We categorized them as diet restrictors and nonrestrictors based on whether they are following any change in dietary pattern depending upon the aggravation of joint symptoms following a specific food intake. We also considered associated comorbidities and alternative medicine intake, which can influence the dietary pattern. Out of a total of 101 patients with an average disease duration of 50.2 ± 65.4 months, 44 (43.6%) followed dietary restriction while 57 (56.4%) did not observe any restriction. 20 of the 44 patients who restricted their diet (45.5%) felt a difference in joint symptoms on exposure to particular diet; 52 out of 57 (91.2%) nonrestrictors did not experience any influence of diet on their symptoms. Patients who observed changes in their joint symptoms on exposure to a particular diet adopted dietary restriction and we observe this association as statistically significant in our study (p <0.0001). 20 out of the 44 and 52 out of 57 patients in either group appeared to be correctly judging whether or not to restrict their diets.

We concluded that the decision of modifying diet should be individual case-based rather than a generalized dietary restriction, which lacks a solid scientific proof as of now and might deprive the individual of the benefits of a nutritious diet. The voluntary addition or deletion of part of the diet might well have an influence on the overall dietary habits of the entire family, as most often RA affects women who are often the main homemaker. Unlike gouty arthritis where there are definite dietary triggers, rheumatoid arthritis is a disease with complex pathogenesis and variable course and hence needs to be managed in a multifactorial manner with the basic recommendation for a healthy and nutritious diet till we have convincing data regarding the benefits of dietary manipulation.

## References

[1] Lourdudoss C., Arnaud L., Wolk A., van Vollenhoven R. F., Di Giuseppe D. (2018). Long-term dietary changes after diagnosis of rheumatoid arthritis in swedish women: data from a population-based cohort. *International Journal of Rheumatology*.

[2] Hagen K. B., Byfuglien M. G., Falzon L., Olsen S. U., Smedslund G. (2009). Dietary interventions for rheumatoid arthritis. *Cochrane Library: Cochrane Reviews*.

[3] Petchiappan V., Priyadarshini P., Prabhu V. N. (2018). Dietary restrictions in rheumatoid arthritis-what the patient?s feel: A questionnaire based study. *Archives of General Internal Medicine*.

